# Neutral evolution test of the spike protein of SARS-CoV-2 and its implications in the binding to ACE2

**DOI:** 10.1038/s41598-021-96950-z

**Published:** 2021-09-22

**Authors:** Georgina I. López-Cortés, Miryam Palacios-Pérez, Gabriel S. Zamudio, Hannya F. Veledíaz, Enrique Ortega, Marco V. José

**Affiliations:** 1 Department of Immunology, Instituto de Investigaciones Biomédicas, Universidad Nacional Autónoma de México, 04510 CDMX, Mexico; 2 Theoretical Biology Group, Instituto de Investigaciones Biomédicas, Universidad Nacional Autónoma de México, 04510 CDMX, Mexico; 3 Universidad Latinoamericana, Nutrición, Campus Cuernavaca, Morelos Mexico

**Keywords:** Epidemiology, Evolution, Medical research

## Abstract

As the SARS-CoV-2 has spread and the pandemic has dragged on, the virus continued to evolve rapidly resulting in the emergence of new highly transmissible variants that can be of public health concern. The evolutionary mechanisms that drove this rapid diversity are not well understood but neutral evolution should open the first insight. The neutral theory of evolution states that most mutations in the nucleic acid sequences are random and they can be fixed or disappear by purifying selection. Herein, we performed a neutrality test to better understand the selective pressures exerted over SARS-CoV-2 spike protein from homologue proteins of *Betacoronavirus*, as well as to the spikes from human clinical isolates of the virus. Specifically, Tyr and Asn have higher occurrence rates on the Receptor Binding Domain (RBD) and in the overall sequence of spike proteins of *Betacoronavirus*, whereas His and Arg have lower occurrence rates. The in vivo evolutionary phenomenon of SARS-CoV-2 shows that Glu, Lys, Phe, and Val have the highest probability of occurrence in the emergent viral particles. Amino acids that have higher occurrence than the expected by the neutral control, are favorable and are fixed in the sequence while the ones that have lower occurrence than expected, influence the stability and/or functionality of the protein. Our results show that most unique mutations either for SARS-CoV-2 or its variants of health concern are under selective pressures, which could be related either to the evasion of the immune system, increasing the virus’ fitness or altering protein – protein interactions with host proteins. We explored the consequences of those selected mutations in the structure and protein – protein interaction with the receptor. Altogether all these forces have shaped the spike protein and the continually evolving variants.

## Introduction

The ongoing COVID-19 pandemic caused by the rapid global transmission of SARS-CoV-2^1^ illustrates the planetary consequences of recurrent episodes of zoonotic transmission from animals to human populations. At least seven coronaviruses have been identified to infect humans causing principally respiratory difficulties but only three of them pose potential pandemic threats^2–4^. Among the 4 genera of coronavirus, only some virus of the *Alphacoronavirus* and the *Betacoronavirus* can infect humans^5^. These two genera have a common ancestor that infects bats while the *Gammacoronavirus* and *Deltacoronavirus* have a bird coronavirus origin^5^. This means that human coronavirus may be directly related to bat coronavirus or to other mammals as intermediate hosts. Phylogenetic analyses have revealed that SARS-CoV-2 is a *Betacoronavirus* related to the bat *Rhinolophus affinis* coronavirus Bat-SL-RaTG13 and the Malayan pangolin (*Manis javanica*) coronavirus, and that SARS-CoV-2 and SARS-CoV belong to the same B lineage, whereas MERS-CoV belongs to the C lineage^1,6^.

Structural and genomic analysis of viral components are key for understanding the evolution of the virus and developing vaccines and therapeutic strategies both to combat the pandemic and to prevent further spread. As all the coronavirus, SARS-CoV-2 recognizes and fuses into the host cells membranes through the spike glycoprotein^7^. The SARS-CoV-2 spike glycoprotein (SARS2-S) attaches to the human Angiotensin Converting Enzyme 2 (ACE2) expressed on the cell membrane and is further processed by host’s proteases^8^ which are necessary for fusion. SARS2-S is made up of the Subunit 1 (S1) that contains the Receptor Binding Domain (RBD) and Subunit 2 (S2), responsible for fusion with the cell membrane^9,10^. Two conformations are described for the SARS2-S where the RBD is in a down position, or in an up position, this last one interacts with the receptor^11^. In the viral membrane, the spike protein interacts with other two spike proteins forming an homotrimer^10,11^. Given the essential role of this protein in the viral life cycle, it is assumed that it has undergone strong evolutionary pressures to ensure the propagation of the virus. The human membrane protease ACE2 has been identified as the viral receptor for several coronavirus, including other bat SARS-like coronavirus, the SARS-CoV and SARS-CoV-2, as well as the *Alphacoronavirus* hCoV-NL63^6,7^. Different analyses revealed that the RBD of spike proteins of SARS-CoV-2, SARS-CoV and MERS-CoV allow binding to the receptor from various species while staying within a range of possible mutations^10,11^, although neither the binding affinities nor the effect of such mutations on the affinity have been quantified. What has been certainly demonstrated through structural analysis and surface plasmon resonance, is that the binding affinity of SARS2-S protein to its receptor ACE2 is greater than the one of SARS-CoV to the same receptor^12,13^. The most probable amino acids responsible for the increase in affinity that could have resulted in enhancing the spread of the virus SARS-CoV-2 have already been proposed^9,14–18^.

Herein, we analyze the reference sequence of the spike protein of the SARS-CoV-2 and compare it to the sequences of spike proteins from other coronavirus, to best fit an evolution model that explains the amino acids preferences that have been selected for a higher affinity binding to the host’s receptor. Furthermore, we demonstrated the differences of the selective pressures exerted over the ongoing evolving virus. To understand the evolution of the spike protein, we applied an amino acid substitution test (neutrality test) to identify the amino acids that deviate from neutral selection. This test is directly related to the degeneracy of the Standard Genetic Code (SGC)^17^ and was applied to both the whole sequence of the spike protein and to the sequence of the RBD as well as to the spikes from human clinical isolates. Then we identified unique mutations in SARS2-S that increased the protein’s binding to its receptor, in terms of their physicochemical properties. Finally, we discuss how the mutations of variants could affect both the protein structure and the success of the virus.

## Materials and methods

### Data sources

The nucleotide and amino acid sequences of the spike proteins of 102 *Betacoronavirus* reported in the GenBank (https://www.ncbi.nlm.nih.gov/) were obtained. In addition, we downloaded 561 whole genome sequences of SARS-CoV-2 from human isolates reported in the GenBank. From the whole genomes, 200 spike sequences were obtained. The structures of the RBDs of SARS2-S in complex with ACE2 (6M0J), SARS-S RBD bound to ACE2 (2AJF), were downloaded from the Protein Data Bank (https://www.rcsb.org/). Also, the reference structure of SARS2-S was downloaded from the SARS-CoV-2-dedicated ZhangLab webpage (https://zhanglab.ccmb.med.umich.edu/COVID-19/). SARS-CoV-2 variants’ spike sequences were retrieved from Situation Reports deposited in the site outbreak.info.

### Neutral evolution model

The SGC has been mathematically modeled into a 6-dimensional (6D) hypercube using group theory^18^. The 6D representation of the SGC means that any codon has 6 other codons to which it is adjacent. The 6D-model has been further transformed into its amino acid phenotypic graph representation^19^. In this graph of the 20 canonical amino acids, the vertices represent the amino acids; and two amino acids (aa1 and aa2) are joined by an edge; at least one codon that encodes aa1 is at one edge distance to at least one codon that encodes aa2 in the 6D-model of the SGC. For a given amino acid, the set of adjacencies of its codons represent the possible changes the amino acid can undergo with a single nucleotide mutation. The normalization by rows of this adjacency matrix leads to a probability transition matrix of a stochastic process whose states are the amino acids. Adding the Markov property to the stochastic process results in a discrete time stochastic process with no memory. The limiting distribution (or stationary probability distribution), of the average transition matrix is a neutral control of the changes present in a protein history at amino acid level. The substitution matrices for each set of protein sequences are a heuristic evolutionary model for each protein. The stationary distributions for each protein reflect the probability of occurrence of each amino acid in the proteins if they continue evolving with their current evolutionary model. Thus, our amino acid neutral test considers all positions as equally likely to change, with the sole constraint that they obey the degeneracy of the SGC. If a component from the stationary distribution, obtained from protein sequences, has a greater value than its corresponding component at the neutral control, it will be interpreted as positive selection, and lower values will be considered as negative selection. The values that lie at or close to the neutral control will be interpreted as consistent with a neutral mutation according to the random drift hypothesis of molecular evolution. This test measures unambiguously the levels of positive or negatively selected amino acids as well as those that are neutral or close to neutrality, without the need of a phylogenetic tree^20^. Our results cannot be directly visualized or measured from a phylogenetic tree.

#### Data

One hundred two spike sequences of *Betacoronavirus* were pairwise aligned. Each pair of protein sequences was aligned using MUSCLE^21^ with default parameters. The protein alignment was used as template to derive a nucleotide alignment that would not have gaps that could split codons. From the nucleotide alignment a table of mutations was computed that account for the total of changes in codons. The table of codon mutations was transformed into an amino acid mutation matrix by adding up the values of the codons for a given amino acid. Hence, this matrix considers synonymous and non-synonymous mutations. The amino acid mutation matrix was computed for every pair of sequences and added up. Then, the matrix was normalized by rows, so that each row adds up to 1, and yields a probability transition matrix. The stationary distribution of the probability transition matrix was derived and compared to the control of neutral evolution as described elsewhere^17^. To assess the statistical robustness of the sample of sequences, a jackknife procedure was applied. The procedure of deriving the stationary distribution from the probability transition matrix of a sample of sequences was repeated to all possible subsets of 50 sequences. A confidence interval of 95% was computed around the stationary distribution derived from the set of sequences. The whole procedure was also applied to the RBD sequences of the same spikes of *Betacoronavirus* and to 200 sequences of the spike protein of SARS-CoV-2.

### Phylogenetic analysis

All the evolutionary analyses were conducted with MEGA X software^21,22^. The multiple alignments of the spike sequences were performed with MUSCLE algorithm. The reference sequence of SARS2-S was compared to both groups i) the most proximal amino acid (a.a) sequences and ii) the ACE2 binding CoVs. Consequently, unique mutations for SARS2-S and conserved residues were identified manually. Potential glycosylation sites were identified in both the linear and structural models.

### Structural analysis

The structures were cleaned to have the most accurate protein and complexes of the RBDs to its receptor. The structural analysis was visualized and analyzed with Chimera^23,24^, and I-TASSER^25–29^. The complex of SARS2-S RBD with the receptor was used to point out unique and conserved residues. Distances between the amino acids involved in the protein – protein interaction were computed. Other parameters like the hydrophobicity and electrostatic potential were calculated for the a.a. in the interface. The same was calculated for the complex SARS-S RBD with the receptor. The number of contacts, number of hydrogen bonds, hydrophobicity and mean distances were compared. Besides, spike mutations of the most prominent variants of SARS-CoV-2 were identified in the three-dimensional model of SARS2-S and the structural models were predicted for each of them with I-TASSER platform. The reference structure was downloaded from the SARSCoV2-dedicated ZhangLab webpage (https://zhanglab.ccmb.med.umich.edu/COVID-19/).

## Results

**Figure 1:**
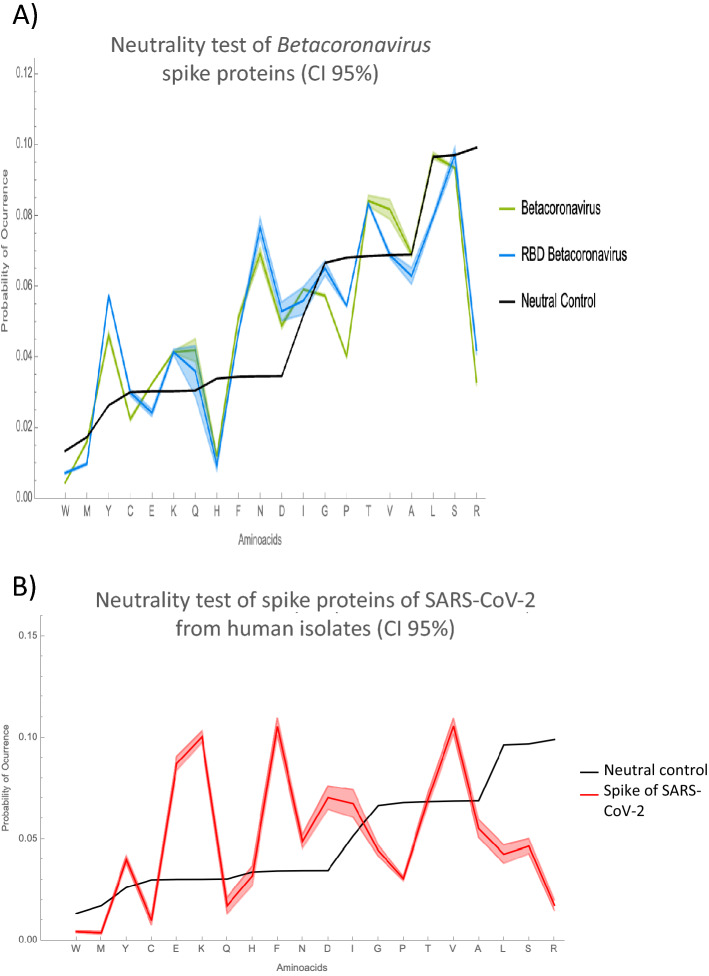
Neutral evolution test of the a.a. of the spike protein and the RBD. The probability of occurrence of each a.a. in a hypothetical protein that has nonselective pressures over its mutations (black line), compared to (**A**) the probability of occurrence for a.a. of the spike protein of *Betacoronavirus* (green) and of the a.a. of the RBD (blue). (**B**) the neutrality test to the spike protein of SARS-CoV-2 from human isolates (red). A.a. with higher occurrence than that predicted by purely stochastic changes are under positive selection pressure, while frequencies lower than the neutral prediction are amino acids that underwent negative selection pressures. A Jackknife procedure was performed to obtain confidence interval of 95%. All calculations were made with Wolfram Mathematica 12.3.

The neutral theory of molecular evolution assumes that evolution is driven by random point mutations that eventually may be fixed by genetic drift or selection. From this point of view, we applied an amino acid neutrality test^17^ to better understand the type of selective pressure, if any, acting upon a.a. present in the spike protein. This analysis revealed that in the *Betacoronavirus* genus, Trp, Cys, His, Gly, Pro, Ser, and Arg underwent negative selective pressures, as the number of changes in these a.a. are lower than the expected by neutral evolution. In contrast, Tyr, Lys, Gln, Phe, Asn, Asp, Thr, and Val, displayed positive selection and Met, Glu, Ala, and Leu are amino acids fixed by genetic drift or neutral forces (Fig. 1A). To accurately analyze the adaptation of SARS-CoV-2 to its receptor, we applied the neutrality test to the RBD of the spike sequences. Importantly, Trp, Met, Glu, His, Pro, Ala, Leu, and Arg showed negative selection, whereas Tyr, Lys, Phe, Asn, Asp, and Thr manifested positive selection in the domain (Fig. 1A). This means that it is less likely to find an Arg that appeared by random mutation than a Tyr or an Asn, because the hexa-codonic Arg is under high negative selective pressure.

To better understand the ongoing evolution of SARS-CoV-2, we performed another neutrality test to the spike proteins of SARS-CoV-2 from human isolates. Figure 1B shows that Tyr, Glu, Lys, Phe, Asn, Asp, Ile, and Val are under positive selective pressure whereas Trp, Met, Cys, Gln, Gly, Pro, Ala, Leu, Ser, and Arg are under negative selective pressures. The differences between graphs of Fig. 1A, B highlights the sharpness of the model to detect the selective pressures in a protein belonging to a lower taxon. Moreover, we performed the neutrality test to the whole genome of 561 sequences of SARS-CoV-2 from human isolates (Figure S1). This analysis permits to portray how the whole genome of SARS-CoV-2 is subjected to selective pressures, which are not necessarily the same along the whole viral genome. Different viral proteins are influenced by different variables that modify the probability of occurrence of their own amino acids.

**Figure 2:**
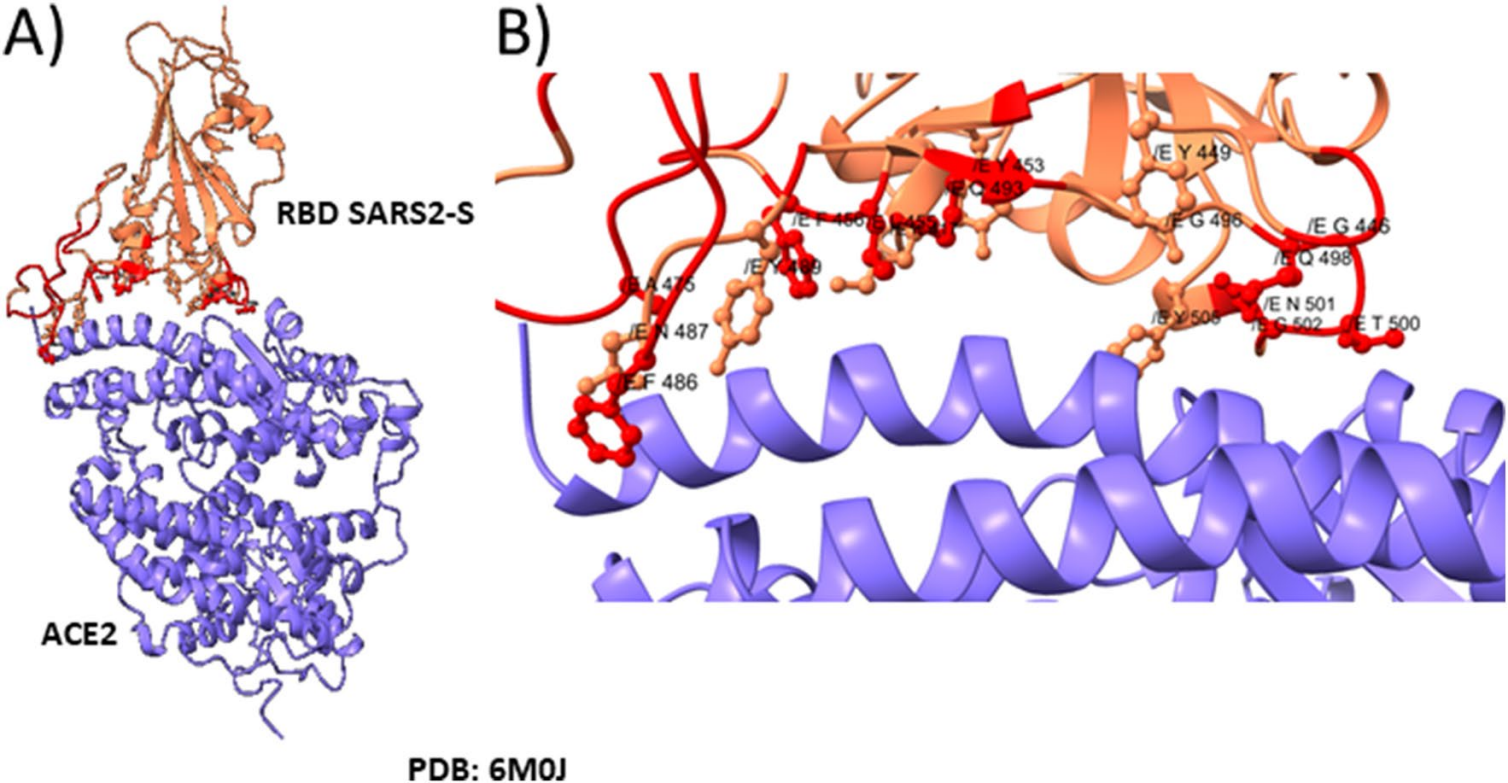
Interaction between the RBD of the spike protein of SARS-CoV-2 with ACE2. (**A**) Interaction between the RBD of SARS2-S (pale pink) and the human receptor ACE2 (blue). (**B**) A close-up of the interface shows the R side chains of the a.a. of the RBD involved in the binding with the human receptor. Unique a.a. for SARS-CoV-2 are colored in red.

**Figure 3:**
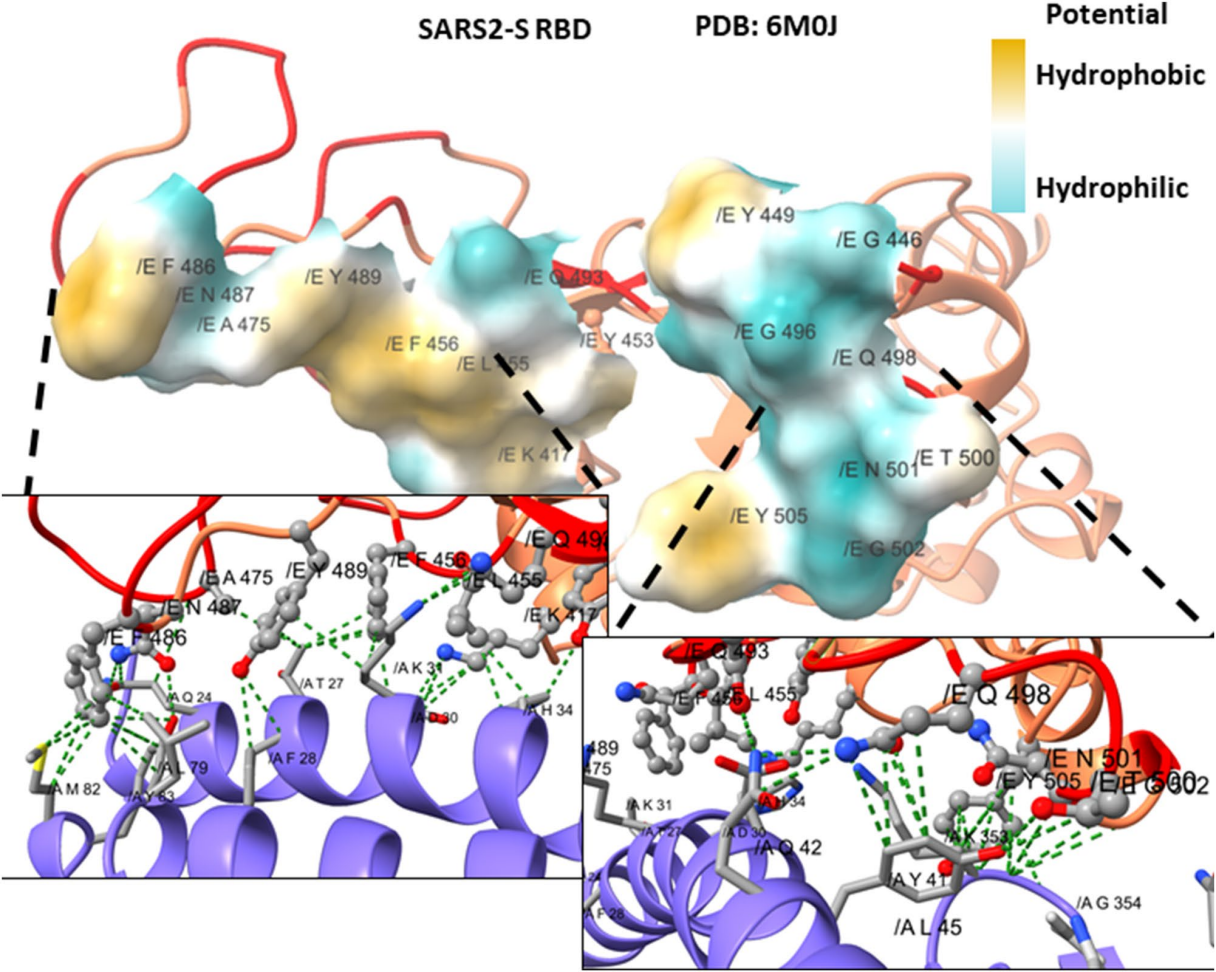
Unique residues of SARS2-S contact more the receptor ACE2. The surface of the amino acids involved in protein–protein interaction with the receptor is colored by its hydrophobic potential. Specific pockets are zoom in to show the responsible residues. Compared to other ACE2 binding spikes, SARS2-S has Phe 456 and Phe 486 in the interface which enables more hydrophobic area (left down) and Gln 498 enables a hydrophilic area, all three establishes more contacts with the receptor compared to other ACE2 binding proteins.

We carried out a multiple alignment of the a.a. sequences of spike proteins of coronavirus including *Alphacoronavirus* and *Betacoronavirus.* Consequently, we identified the most related spike sequences to SARS-CoV-2 (not shown). We focused in the RBD sequence of the S1 subdomain to identify mutations that could be advantageous for SARS2-S binding to ACE2. Therefore, another multiple alignment was performed including the spikes of SARS-CoV-2, RaTG13, PnCoV, and several spikes known to bind to human ACE ^8^. We discarded the *Alphacoronavirus* HCoV NL63 because even though it binds to the human ACE2, the orientation of the RBD in the protein – protein interaction is different. We noticed 49 unique mutations in SARS2-S compared to the others ACE2 binding sequences (Table S1). Some of these mutations are present in CoV RaTG13 and PnCoV spikes, what would suggest that these spike proteins could bind to the human ACE2 as well. Those mutations were localized in the structure of the complex with ACE2 (PDB: 6M0J). Interestingly, most mutations are located at the interface with ACE2 (Figure S2). Figure 2A shows the interaction between SARS2-S RBD and ACE2 (modified from PDB: 6M0J), all the unique amino acids are colored in red. Figure 2B shows a close-up where the side chains of the amino acids involved in the protein–protein interaction are shown with sticks. We compared the conservation of the residues among all the ACE2 binding sequences. The conserved residues are shown in pale pink as the rest of the structure (i.e., Tyr 449, Tyr 453, Asn 487, Tyr 489, Thr 500, Gly 502, Tyr 505) (Table 1) while the unique a.a. for SARS2-S, are shown in red (Fig. 2B). Among the 17 a.a. involved in the interaction, 10 are unique for SARS2-S, including Lys 417, Gly 446, Leu 455, Phe 456, Ala 475, Phe 486, Gln 493, Gly 495, Gln 498 and Asn 501 (Table 2). We analyzed unique a.a. that raised affinity to the receptor, therefore were selected during the evolutionary process. The mutations Gly 446 and Lys 417 enable the formation of new hydrogen bonds with the receptor, SARS-S forms 8 hydrogen bonds with the receptor while SARS2-S forms 11 (Supplementary Material). Specifically, Phe 456 and Phe 486 that are under positive selection were selected because they generate more hydrophobic contacts with the receptor than the Leu’s at the same positions present in SARS-S. At the other edge of the interface, Gln 498 which was selected neutrally in the RBD enables a hydrophilic surface that permits interactions with the receptor by van der Waals forces (Fig. 3).

**Table 1:**
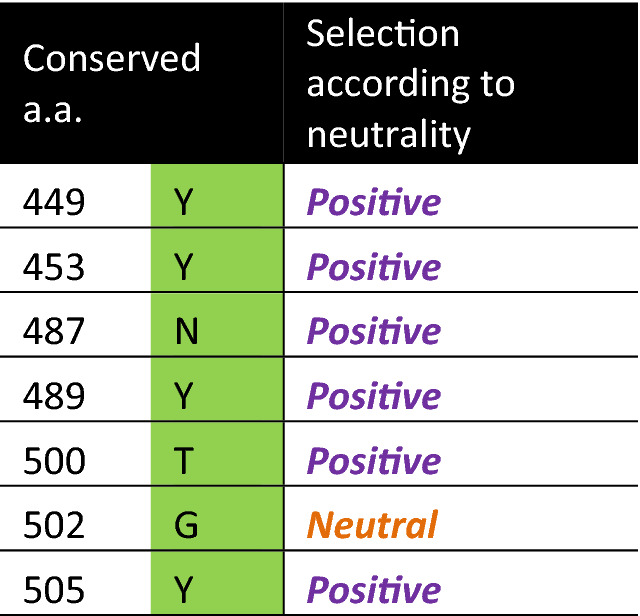
Conserved residues involved in protein–protein interaction with ACE2 among the ACE2 binding spikes.

The selection type according to the neutrality test are indicated.

.

**Table 2:**
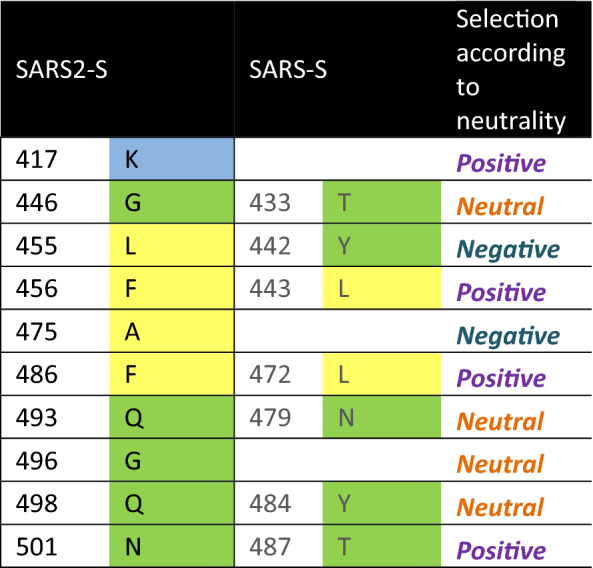
Unique residues for SARS2-S involved in protein–protein interaction with ACE2.

The selection type according to the neutrality test are mentioned specifically for SARS2-S. Other ACE2 binding spike proteins expressed different a.a. Here SARS-S is shown as an example. The empty spaces in SARS-S are a.a. that do not interact with ACE2.

.

**Figure 4:**
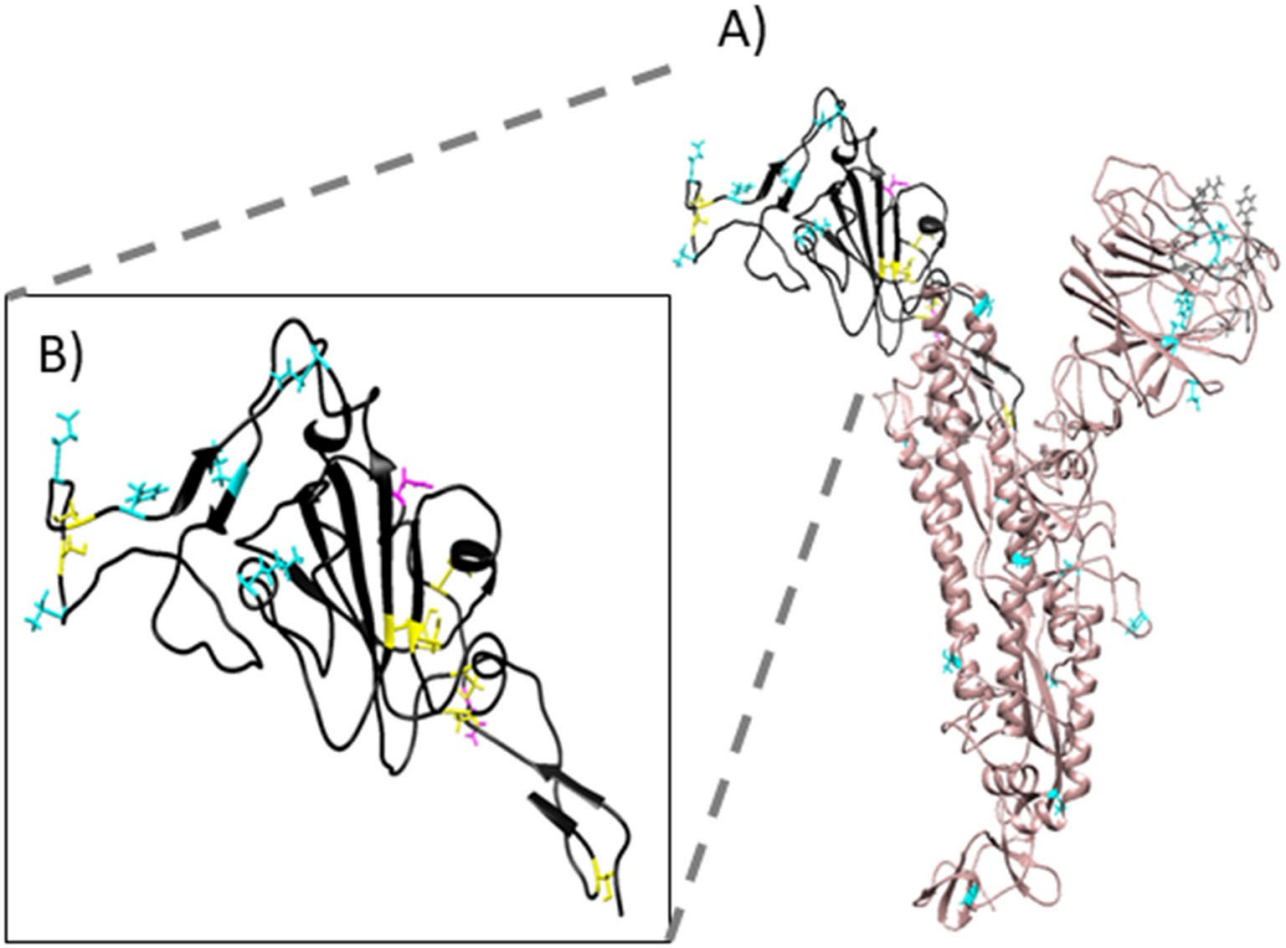
Structure of the spike protein of SARS-CoV-2 and the variants of concern. (**A**) Structure of the spike protein of SARS-CoV-2 in rosy-brown color. (**B**) Zoom of the RBD structure of SARS2-S colored in black. Cys of the RBD are shadowed in yellow and the two glycosylated Asn are magenta. All sites of point mutations in the variants (from Alpha to Lambda) are shadowed in cyan and deletions in grey. Molecular graphics and analyses performed with UCSF Chimera, developed by the Resource for Biocomputing, Visualization, and Informatics at the University of California, San Francisco, with support from N1H P41-GM103311.

Then we concentrated in the mutations of spikes proteins of SARS-CoV-2 variants to unravel the evolutionary behavior of the virus. The variants that have received most attention due to their importance for public health are those identified as variants of concern and variants of interest. To know if the mutations have modified the structure of spike of some variants, we downloaded the reference structure of SARS2-S from I-TASSER and modified to point all mutations in the variants in cyan and deletions in grey (Fig. 4A); the RBD is magnified in the inset (black) (Fig. 4B). Also, the Cys of the RBD are shadowed in yellow and the two glycosylated Asn are in magenta (Fig. 4B). Figure S2 shows the predicted structures of the four variants of health concern: Alpha, Beta, Gamma and Delta as well as three of interest: Epsilon, Iota, and Kappa; they are all merged with the reference structure. To note, the predicted structure of Gamma and Epsilon variants protrude the RBD at a different position than that in the reference structure and the rest of the variants. From the reference structure of SARS2-S downloaded, Alpha deviates only 2.52 Å, Beta 1.97 Å, Gamma 4.14 Å, Delta 3.34 Å, Epsilon 4.2 Å, Iota 3.21 Å and Kappa 3.23 Å (Root Mean Square Deviation). Then, we identified the selective pressures over each mutation in the spike proteins of the variants mentioned above, and four more variants of interest (Zeta, Eta, Theta and Lambda). Interestingly, all variants share the mutation D614G, three of the variants of concern and one of interest presents N501Y substitution (Table 3). Also, the Beta, Gamma, Zeta, Eta and Teta share E484K, and the Delta, Epsilon and Kappa variants share L452R. Tyr and Lys are positively selected a.a. both for the complete spike proteins as well as for the RBDs of *Betacoronavirus* and SARS-CoV-2 human isolates. In contrast, Gly and Arg were clearly negatively selected at position 614 and 452 respectively (Fig. 1). Gamma, Zeta, and Theta present a Phe in position 1176 and the Pro 681 is often substituted either by His or Arg.

**Table 3:**
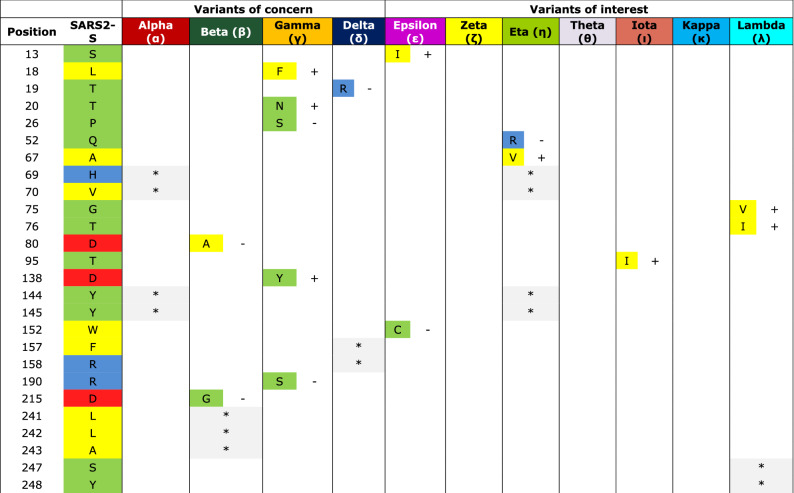

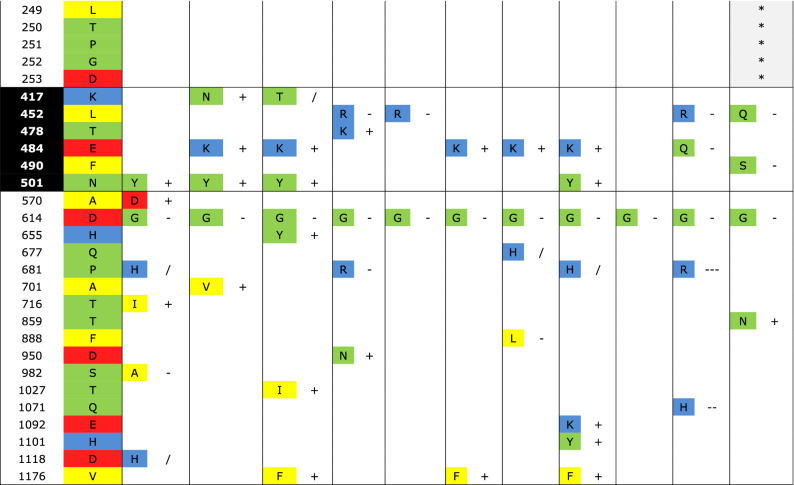
Type of selective pressure for mutations in health concern variants.

The mutations colored by physicochemical properties are enlisted for each variant and the type of selective pressure applied for each a.a. is shown. The type of pressure is specified either for the whole protein or for amino acids positioned in the RBD.

"Position" column is according to the RefSeq.

The substituting amino acid on each variant is indicated in one-letter code next and the selective pressure exerted over the a.a. according to the neutrality test. Positive (+), negative (−), neutral (/).

Asterisks indicate deletion.

.

Finally, as shown recently, SARS2-S is highly glycosylated which forms a protective shield against recognition of the immune system. Hence, we considered the conservation of the glycosylation sites important for evolution of the virus. The glycosylation sites reported for SARS2-S^29^ were identified and compared to the other ACE2 binding sequences (Table 4). Four Asn (17, 74, 149 and 657) are unique to SARS2-S, while the rest (18) are shared and from the three a.a. that forms O-glycosilations, only one is conserved. To note, PgCoV and bat CoV RaTG13 express all potential glycosylation sites identified in the sequence of the causal agent of COVID-19, meaning that those a.a. were selected even before SARS-CoV-2 infected humans. Nevertheless, they all contribute to avoid the recognition of the immune system without affecting the binding to the receptor ACE2. We also identified mutations close to glycosidic sites in the SARS-CoV-2 variants of concern. All of them possess mutations near to these sites.

**Table 4:** Conservation of the glycosylation sites of SARS2-S. Most of the glycosylation sites reported from SARS2-S are shared with all the ACE2 binding sequences except for 6 residues 4 Arg, 1 Thr and 1 Ser.

SARS2-S	Glycosylation	Conserved	Variants with mutations near by	SARS2-S	Glycosylation	Conserved	Variants with mutations near by
17	N-gly	No	γ, δ	616	N-gly	Yes	α, β, γ, δ, ε, ζ, η, θ, ι, κ, λ
61	N-gly	Yes	α, η	657	N-gly	No	γ
74	N-gly	No	α, η, λ	676	O-gly	Yes	η
122	N-gly	Yes		709	N-gly	Yes	β
149	N-gly	No	α, η	717	N-gly	Yes	α
165	N-gly	Yes		801	N-gly	Yes	
234	N-gly	Yes		1074	N-gly	Yes	κ
282	N-gly	Yes		1098	N-gly	Yes	
323	O-gly	No		1119	N-gly	Yes	α
325	O-gly	No		1134	N-gly	Yes	
331	N-gly	Yes		1173	N-gly	Yes	γ, ζ, θ
343	N-gly	Yes		1194	N-gly	Yes	
603	N-gly	Yes					

All variants of concern present mutations near at least one unique glycosylated residue.

## Discussion

The neutral theory of evolution states that most mutations in nucleic acid sequences are random, and these can be fixed by genetic drift and/or natural selection. We exploited an amino acid neutrality test to interrogate the molecular evolution of the spike protein and to consider the possible implications for binding to its receptor that is why we focused on the RBD sequences. Positive selective pressures cause a.a. to be fixed in higher frequencies than by neutral evolution or genetic drift, while negative selective pressures cause a.a. to appear in lower frequencies than the neutral control. Fixed mutations under negative selective pressures may remain because they are advantageous for the virus by contributing to the protein stability or increasing the affinity for its receptor. These selective pressures are similar among homologous proteins and importantly, they are the major driving forces for adaptation.

The neutrality test applied to the RBD sequences, shows that there are amino acids that have similar selective pressures as the whole protein, with few notable exceptions. For example, Tyr is subject to a higher positive selection in the RBD than in the whole protein; actually, four Tyr are involved in protein – protein interaction with the receptor. In fact, most common residues between the ACE2 binding proteins have positive selection. However, unique mutations in SARS2-S RBD were fixed by selective pressures rather than neutral forces. Hence, the adaptability of this domain is explained by quantitative means. Specifically, the mutations that contributed to a higher affinity to the receptor, compared to SARS-S, were also identified. Phe 456 and Phe 486 generate more hydrophobic interactions with atoms from ACE2, Gln 498 enhances interaction with the receptor by van der Waals forces, and Lys 417 enabled the formation of new hydrogen bonds with the receptor. These mutations certainly were selected for increasing the affinity with the receptor.

Besides spike proteins need to be processed by different host proteases to disassemble S1 from S2 and to expose the fusion peptide, consequently the fusion machinery could induce the membranes fusion and thus to achieve the cell infection. Other coronavirus are cleaved by trypsin, furin-like convertases, cathepsins, serin proteases, elastases, plasmin, among others, in order to achieve the fusion peptide exposure^30^. Thus, host proteases exert an important selective pressure to the spikes proteins and are responsible for tropisms toward cells who express them. The fact that the polybasic motif (682-RRAR) in SARS2-S resulted from an insertion of 12 nucleotides (681-PRRA), has generated the COVID lab-leak hypothesis, a claim that a coronavirus was manually manipulated in a laboratory and sparked doubts about the origin of SARS-CoV-2. However, others have discussed that this motif has appeared multiple times and independently in spike proteins of others coronavirus^31^. It is not clear whether there was an epistasis event that enables the 4 amino acid insertion but certainly the motif was selected because it increases the pathogenicity of the virus^32^. The cleavage not only disrupts the binding between S1 and S2 and approaches the fusion machinery, but also generates a neuropilin-1 binding motif^33,34^ which contributes to the internalization of the virus^35^. Within the context of the neutral theory of evolution, the probability of occurrence of each amino acid is related to their codonicity; in other words, since Arg is hexa-codonic, and Pro and Ala are tetra-codonic, the probability of appearance of these specific amino acids turns to be high. According to our results, the probability of occurrence of Pro and Arg is lower than the expected in the whole protein. Nevertheless, once presented, they were selected because they were part of a motif that gave a specific advantage to the virus. So, the expression of a polybasic motif at S1′/S2′ which is susceptible to a specific protease should not be regarded as a surprise. Other coronavirus that present similar polybasic motifs in the S1′/S2′ site, where shown in our multiple alignment of spikes of *Betacoronavirus*: the spikes of the Murine Hepatitis Virus (YP009824982.1), Murine CoV RA59/R13 (ACN89689.1), Murine CoV RA59/SJHM (ACN89705.1), Rat CoV (YP003029848.1), HCoV HKU1 (YP173238.1), HCoV OC43 (YP009555241.1), Rabbit CoV (YP005454245.1), Canine CoV (AQT26498.1), Human enteric CoV (ACJ35486.1), Bovine CoV (NP150077.1) and the Sambar deer CoV US/OH-WD388TC/1994 (ACJ67012.1).

Herein, we present a model that analyzes the preference of amino acids by means of the selective pressures that enhances transmissibility or increase the stability of the interaction with the receptor. The substitutions at position 681 may cause slight differences in the secondary structure immediately before the furin cleavage site since Pro introduces slight bends to the protein structure. We certainly calculated the predicted structure of the protein which is very similar to the known structure. Only the Gamma and Epsilon variants had a predicted structure slightly different from the reference. Further predictions should be tested to know if there are greater differences in the trimer and if the interaction with the receptor is enhanced in any spike variant. Regarding this site, it is predicted by computational analysis that the mutation P681R increased the basic properties of the motif, which in turn increased the affinity to the furin enzymatic site^36^. In fact, the mutation does augment the fusogenic properties and enhances syncytia formation. It seems that this substitution permits a Low Complexity Region that contributes to immune system evasion by increasing antigenic variability^37^.

The neutrality test performed to the spike protein of SARS-CoV-2 from human isolates shows the current evolutionary behavior of the virus. We could apply the test mainly because the fast spread of the virus increased its population size that allowed the appearance of mutations. Here we demonstrated in a quantitative manner the actual selective pressures exerted over the virus. Figure 1B shows that some of the positive selected a.a. for spike proteins in *Betacoronavirus* are still under positive selection in the spike proteins of SARS-CoV-2 (i.e. Tyr, Lys, Phe, Asn, Asp), although the probability could be altered.

By the neutrality test we observed that the common mutations present in different variants of concern were fixed by common selective pressures. For example, the Beta and Gamma variants substitute a Lys at position 417. It remains to confirm if these spikes maintain the hydrogen bond formed in the ancestral sequence as Asp has no electron donor. The mutation E484K, present in the same variants and others three, occurs towards an a.a. with almost the same polar requirement  ^45,46^ and it is strongly positive selected. This shared mutation leads to neutralizing antibodies resistance^38^. Other shared mutation is D614G substitution which is present in all variants analyzed. The mutation translates to a small a.a. without charge that is negatively selected. It has been proven that this substitution enhances the binding to ACE2 in comparison with the ancestral virus, therefore infectivity and transmission of the variant also has raised^47–50^. In fact, patients carrying virus with this mutation, have a significantly higher viral load^39^. D614G also alters the conformation of the RBDs towards the up position, which is necessary for receptor recognition^40^. Besides, N501Y substitution another shared mutation increases the binding to the receptor^41,42^. Actually this substitution enables infection to mice cells through interaction with mouse ACE2^51^.

Shared mutations of the variants of concern are reported to increase transmissibility and or favor a conformational state change^40^. The strong selective pressure exerted on these substitutions has already driven them to fixation in SARS2-S. Shared mutations among different health concern variants arose by convergent evolution coming from selective pressures regarding the viral fitness by increasing the pathogenicity, protein–protein interaction, and the evasion of the immune system^43^. However, another selective pressure comes from other viral particles if the viral particles are constantly competing between them. By now Delta variant has displaced rapidly the Alpha variant, which was the predominant variant in UK and USA^44^.

The accumulation of mutations is linked to the capability to correct errors. Coronavirus have RNA-dependent RNA polymerases which are prone to mistake, unlike other RNA virus, they have also a 3′ to 5′ exoribonuclease (nsp14-ExoN) that proofreads the new sequence^41^. Nsp14-ExoN is one of the major factors enabling long and stable RNA genomes, nevertheless some mutations scape from the proofreading. Mutations in the S2 subdomain would probably be less frequent to reach fixation because the sequence is crucial for the function. This subdomain is involved in fussing with the membrane, so the domains require specific physicochemical characteristics. Therefore, purifying selection deletes most neutral mutations, and consequently, there is a high degree of conservation. On the contrary, S1 is important for receptor recognition, specifically the Carboxyl Terminal Domain (CTD) where the RBD is contained, so the sequence of this domain is crucial for adapting to the actual host or for the ability to infect other species. Interestingly, some variants have more mutations in S2 whereas others present a few. The exact biological significance of this observation is not clear. It is probable that the variants with few mutations could either i) have been mutating before and by the time they were reported they may have had enough time to fix or disappear neutral mutations at S2, or ii) they may have appeared recently and have mutated only in S1. Now we cannot sustain any hypothesis because we discussed only eleven variants and we lack the accurate mutation rate of each variant and the transmission rate at different places.

Evidence shows the influence of glycosylation in the success of SARS-CoV-2^28,42^, so we included this characteristic in the study. In other coronavirus the highly glycosylated Amino Terminal Domain (NTD), plays an important role in attachment to the host cell and immune response evasion. For influenza C virus and some coronavirus (HCoV HKU1, and HCoV OC43), attachment through 9-O-acetylated sialic acid receptors is crucial and constitute another species barrier^52–55,50^. The interaction with specific hosts’ proteins facilitates the approaching of the fusion machinery to the cell membrane. Furthermore, saccharides mask potential epitopes recognized by antibodies, making it difficult to the immune system to eliminate the virus. It has been observed that the spike glycoprotein must be shielded by the protective glycans from the immune system^29,50^. For the spike protein, Asn mutations are subjected to positive selection both in the RBD and in the whole protein of *Betacoronavirus*. This favors the fixation of new Asn susceptible to glycosylation, only if the sequon NXT/S is expressed too (N stands for Asn, X is any a.a. except Pro, and the third position stands for Thr or Ser). Therefore, the four new glycosylation sites were enhanced by positive selection. It remains to be determined whether the mutations near glycosylation sites interfere with the formation of glycosidic bonds in the variants of health concern. We found that all variants present mutations near glycosylation sites, which directly alter the epitopes recognized by antibodies they have been reported reduce recognition by antibodies^29^, by creating new epitopes. Hence, evasion to the immune system recognition is an important selective pressure that has influenced the fixation of neutral mutations. It is important to undercover to what extent the glycosylation of SARS-CoV-2 spike protein could help us understand the physiopathology of the virus and to develop better prophylactic or therapeutic strategies, effective against all variants.

The neutrality test constructed here considers both synonymous and non-synonymous mutations, which allowed us to obtain information of the types of selective pressures that influenced deviation in fixation from neutral mutations. The limitation of our study is that we cannot detect mutations fixed by epistasis or originated by recombination or concomitant mutations. Therefore, the common mutations are considered only as the result of natural selection or genetic drift. Another consideration is that the neutrality test showed that the amino acids with the capability to be glycosylated are favored but we cannot certainly conclude that specific residues are bonded to a carbohydrate.

## Conclusion

The long-lasting pandemic, the wide geographic distribution, and the rapid contagiousness mainly during epidemic waves, have influenced the generation of variants of SARS-CoV-2. At global or local scale, the evolution of this virus can be appreciated. Vaccines and drugs have been developed and tested aiming to stop transmission which would also result in preventing the virus from mutating and developing new variants. Therefore, evolutionary studies play an important role in the prevention of epidemiological catastrophes and in the development of better treatments that covers most viral variants. The neutrality test computed shows the type of selective pressure for each amino acid in the spike protein of *Betacoronavirus*. This evolutionary study enables to understand and describe changes in SARS2-S sequence that affects its stability, structure, or function. The neutrality test could be exploited more even in SARS2-S sequences to better appreciate the evolutionary behavior of SARS-CoV-2. Further analysis should be done to know whether if the predicted structures of the variants enhance the binding with the receptor and if the interactions in the trimer are not disturbed. In the end mutations have allowed SARS-CoV-2 to become a persistent threat to mankind, on the scale of a pandemic.

## Supplementary Information

Supplementary Information.
