# Supplementary material for: Neutral evolution test of the spike protein of SARS-CoV-2 and its implications in the binding to ACE2
Source: Sci Rep. 2021 Sep 22;11:18847. doi: 10.1038/s41598-021-96950-z (PMC8458503; doi:10.1038/s41598-021-96950-z)

Neutral evolution test of the spike protein of SARS-CoV-2 and its implications in the binding to ACE2

Georgina I. López-Cortés, Miryam Palacios-Pérez, Gabriel S. Zamudio, Hannya F. Veledíaz, Enrique Ortega, Marco V. José

Supplementary Material

Table S1. Unique mutations in the RBD for SARS-CoV-2. Compared to the other ACE2 binding spikes, SARS-CoV-2 spike protein has 49 mutations in the RBD, the majority selected by a positive pressure. The point mutations of the SARS-CoV-2 spike RBD are listed with the corresponding amino acid expressed in the rest of the Spike proteins that bind to ACE2; the amino acids are highlighted depending on the chemical nature: in yellow the non-polar, in green the polar and neutral amino acids, in blue the positively and in red the negatively charged amino acids. Amino acids in contact with ACE2 are in bold type letters.

	**ACE2 binding CoVs**		**SARS- CoV2**			**Chemical changes/ characteristics**
	**Position**	**a.a.**	**Position**	**a.a.**	**Selective pressure**	
	335	P	348	A	*Negative*	Polarity
	341	E	354	N	*Positive*	Charge
	359	T	372	A	*Negative*	Polarity
	360	F	373	S	*Neutral*	Polarity
	371	A	384	P	*Negative*	Polarity
	380	S	393	T	*Positive*	Polar
	389	V	402	I	*Neutral*	Non- polar
	390	K	403	R	*Negative*	Same charge (+)
	393	D	406	E	*Negative*	Same charge (-)
	404	V	**417**	**K**	*Positive*	Polarity and charge
	417	M	430	T	*Positive*	Polarity
	421	L	434	I	*Neutral*	Non- polar
	425	T	438	S	*Neutral*	Polar
	426	R	439	N	*Positive*	Charge
loop 1	428	I	441	L	*Negative*	Non- polar
	430	A	443	S	*Neutral*	Polarity
	431	T	444	K	*Positive*	Charge
	432	S/Q	445	V	*Neutral*	Polarity
	**433**	**T**	**446**	**G**	*Neutral*	Polar
	439	K	452	L	*Negative*	Polarity and charge

	**442**	**Y/S**	**455**	**L**	*Negative*	Non- polar
	**443**	**L**	**456**	**F**	*Positive*	Non- polar
	445	H	458	K	*Positive*	Same charge (+)
	446	G	459	S	*Neutral*	Polar
	447	K	460	N	*Positive*	Charge
	449	R	462	K	*Positive*	Same charge (+)
	457	N	470	T	*Positive*	Polar
	458	V	471	E	*Negative*	Polarity and charge
	459	P	472	I	*Neutral*	Polarity
	460	F	473	Y	*Positive*	Polarity
	461	S	474	Q	*Neutral*	Polar
loop 2	462	P	**475**	**A**	*Negative*	Polarity
	463	D	476	G	*Neutral*	Charge
	464	G	477	S	*Neutral*	Polar
	465	K	478	T	*Positive*	Charge
	467	T	481	N	*Positive*	Polar
	469	P	482	G	*Neutral*	Polar
	470	P	483	V	*Neutral*	Polarity
			484	E	*Negative*	Insertion
	471	A	485	G	*Neutral*	Polarity
	**472**	**L**	**486**	**F**	*Positive*	Non- polar
loop 3	476	W	490	F	*Positive*	Non- polar
	**479**	**N**	**493**	**Q**	*Neutral*	Polar
	480	D	494	S	*Neutral*	Charge
	**484**	**Y**	**498**	**Q**	*Neutral*	Polarity
loop 4	485	T	499	P	*Negative*	Polar
	**487**	**T**	**501**	**N**	*Positive*	Polar
	489	I	503	V	*Neutral*	Non- polar
	505	N	519	H	*Negative*	Charge
a.a. code		Polar	Non-polar	Polar positive	Polar negative	

Figure S1 Neutral evolution test of the a.a. of the whole genome of SARS-CoV-2.

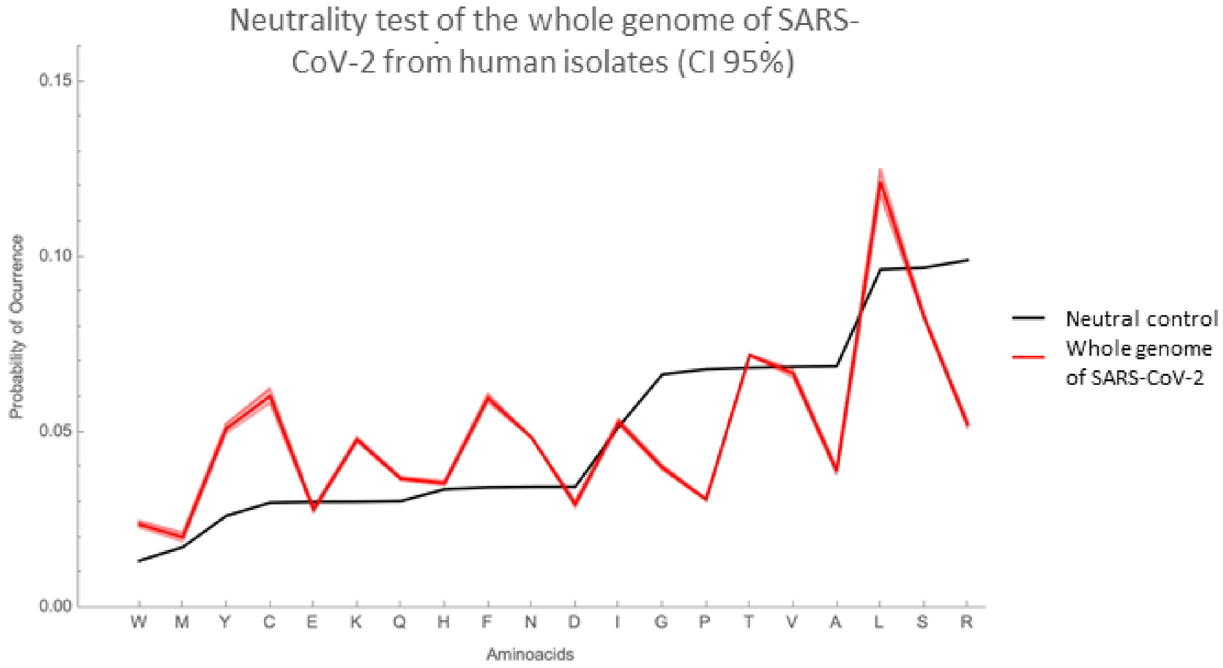

Figure S2 Protein-protein interaction between RBD of SARS2-S and ACE2. **A)** Tridimensional structure of the RBD (red) of the Spike of SARS-CoV-2 interacting with ACE2 (blue) and **B)** the linear representation of spike protein showing the location of the RBD. Each loop in contact with the receptor is colored as in Table S1.

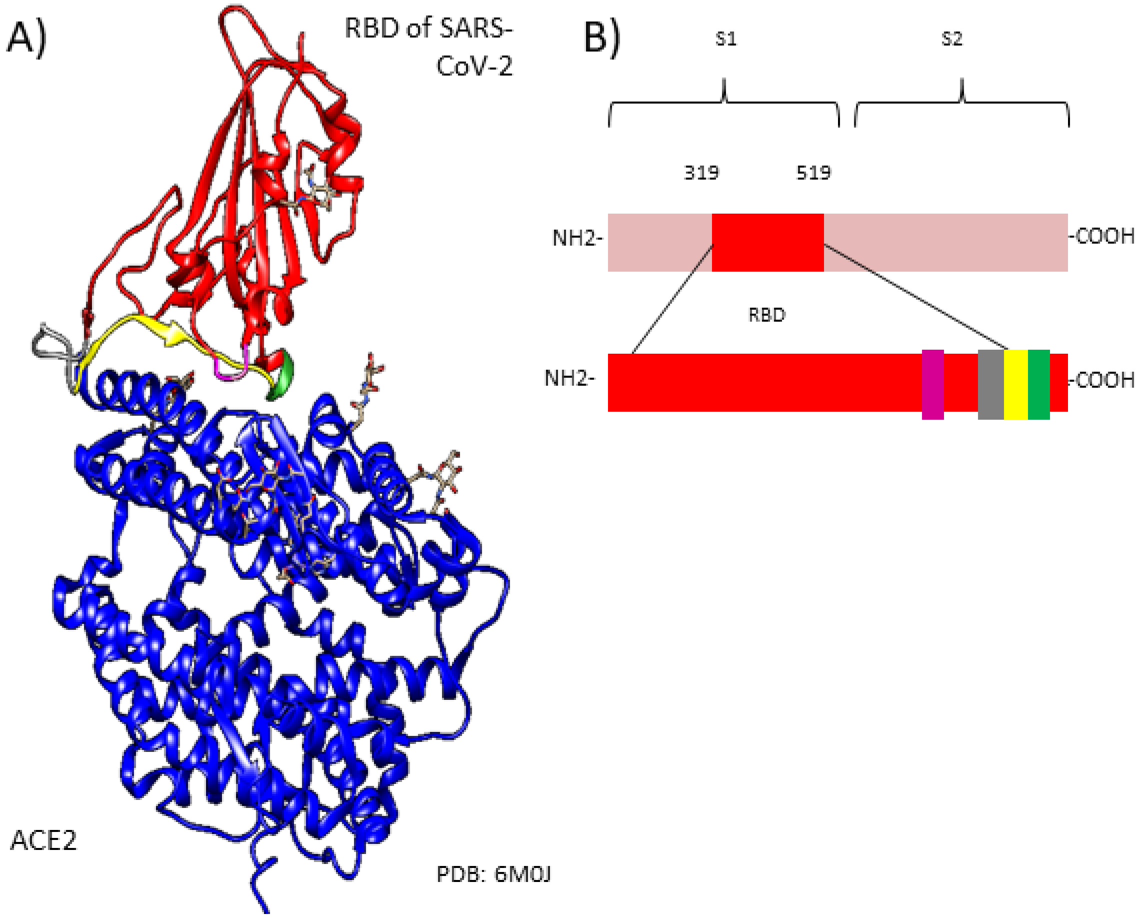

Figure S3. Predicted structures of SARS-CoV-2 variants and superposition of variants onto the reference structure. **A)** Alpha variant in ruby red, **B)** beta variant in dark green, **C)** Gamma variant in goldenrod, **D)** Delta variant in blue navy, **E)** Epsilon variant in dark magenta, **F)** Iota variant in salmon, **G)** Kappa variant in dodger blue. **H)** The reference structure overlapped with the predicted structure of 4 variants of concern, and **I**) The reference structure overlapped with the predicted structure of 7 variants in total. Molecular graphics and analyses performed with UCSF Chimera, developed by the Resource for Biocomputing, Visualization, and Informatics at the University of California, San Francisco, with support from N1H P41-GM103311.

S2. A) Alpha variant, isolated first in United Kingdom

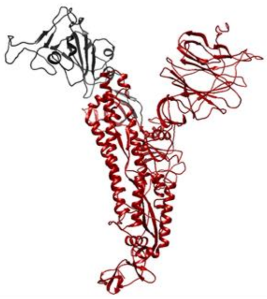

S2. B) Beta variant, isolated first in South Africa

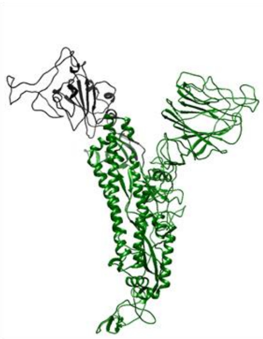

S2. C) Gamma variant, isolated first in Brazil

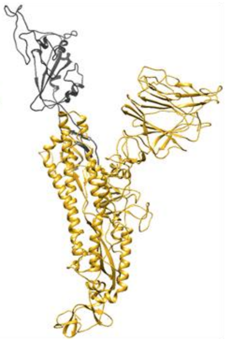

S2. D) Delta variant, isolated first in India

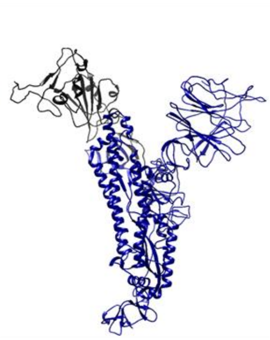

S2. E) Epsilon variant, isolated first in California, USA

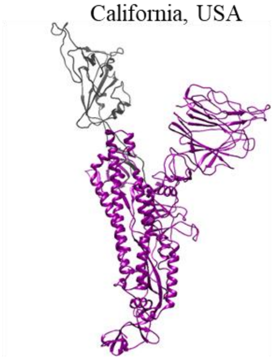

S2. F) Iota variant, isolated first in New York, USA

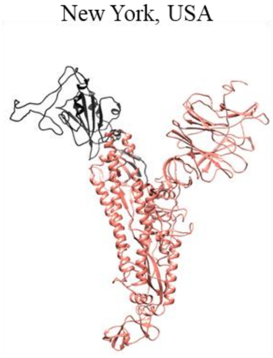

S2. G) Kappa variant, isolated first in India

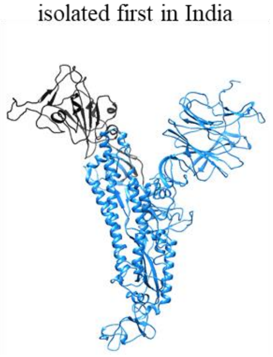

S2. H) The reference structure overlapped with the predicted structure of 4 variants of concern.

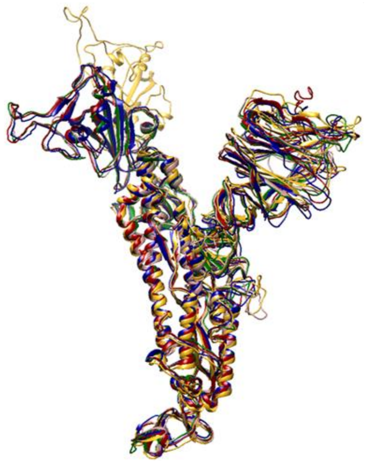

S2. I) The reference structure overlapped with the predicted structure of 7 variants in total.

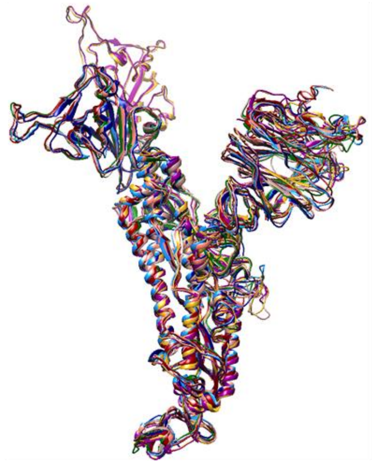

Supplement: Supplementary file 1 — Supplementary Information. [file 41598_2021_96950_MOESM1_ESM.pdf]
